# Intradural Extramedullary Nerve Sheath Myxoma of the Cervical Spine: A Case Report and Review of Literature

**DOI:** 10.3389/fsurg.2021.722254

**Published:** 2022-01-07

**Authors:** Fangfang Xu, Ying Jin, Qian Li, Fei Dong, Liangji Lu, Qingfeng Cui, Chao Wang

**Affiliations:** ^1^Department of Radiology, The Second Affiliated Hospital, Zhejiang University School of Medicine, Hangzhou, China; ^2^Department of Rehabilitation in Traditional Chinese Medicine, The Second Affiliated Hospital, Zhejiang University School of Medicine, Hangzhou, China

**Keywords:** extramedullary tumor, nerve sheath myxoma, spinal tumor, magnetic resonance imaging, diagnosis

## Abstract

**Background:** Nerve sheath myxoma is a rare benign soft tissue tumor. Intraspinal nerve sheath myxomas are rare. Only 8 cases of intraspinal nerve sheath myxoma have been reported to date, and no case of nerve sheath myxoma has been reported in the cervical spinal canal. Herein, we reported the first case of intradural extramedullary nerve sheath myxoma in the cervical spinal canal of a 57-year-old man, including its complete clinical course and radiological findings.

**Case Presentation:** A 57-year-old male patient presented with numbness in his left finger without any obvious inducement for 3 years. CT and contrast-enhanced magnetic resonance imaging (MRI) of the spine were performed. Based on the radiological examinations, a diagnosis of schwannoma was initially made. However, nerve sheath myxoma was finally confirmed by histopathological and immunohistochemical examinations. Complete tumor excision at the C1-2 level was performed. Then, the patient recovered well, and the numbness of his left finger disappeared during the later follow-up after the surgery.

**Conclusion:** Nerve sheath myxoma should receive diagnostic consideration for an extramedullary subdural lesion that is a clear boundary mass characterized by isointensity on T1-weighted images, heterogeneous intensity on T2-weighted images, obvious peripheral enhancement, and a growing tendency toward the intervertebral foramen.

## Introduction

Nerve sheath myxoma was firstly described by Harkin and Reed in 1969, which is a rare benign tumor of nerve sheath originating from the dermis and subcutaneous tissues (1). In 2005, pathologist John Fetsch described 57 cases of nerve sheath myxomas, among which 86% occurred in the extremities, and most commonly included the hands, fingers, knees, pretibial region, ankles, and feet. Only 12.3% of the patients were affected in the trunk or head-neck region (2). Intraspinal nerve sheath myxomas are rare. To the best of our knowledge, only 8 cases of intraspinal nerve sheath myxoma have been reported to date (3–7). However, no case of nerve sheath myxoma has been reported in the cervical spinal canal. Herein, we reported the first case of intradural extramedullary nerve sheath myxoma at the C1-2 level in a 57-year-old man. In this case report, we give a comprehensive description of intraspinal nerve sheath myxoma, including its complete clinical course and radiological findings. Besides, the available literature is reviewed.

## Case Presentation

The study was approved by the Ethics Review Committee of the Second Affiliated Hospital of Zhejiang University School of Medicine. Written informed consent was obtained from the patient for publication of this case report.

### Clinical History

A 57-year-old male patient presented with numbness in his left finger without any obvious inducement for 3 years, which worsened for 4 months. He presented with left occipital pain for 1 1/2 years and slight numbness in the right toe for 3 months. The patient had no nausea, vomiting, chest sulking, shortness of breath, cough, sputum, or other discomforts. His right hand was resected because of trauma 25 years ago. He denied a history of hereditary disease in his family. He was admitted to the Department of Osteology of the Second Affiliated Hospital of Zhejiang University School of Medicine because of worsening symptoms. Physical examination showed decreased shallow sensation of the left upper limb but a normal deep and compound sensation of the left upper limb. Routine blood laboratory tests, including full blood count, renal and liver function tests, were all within normal range.

### Radiological Examinations

**Figure 1:**
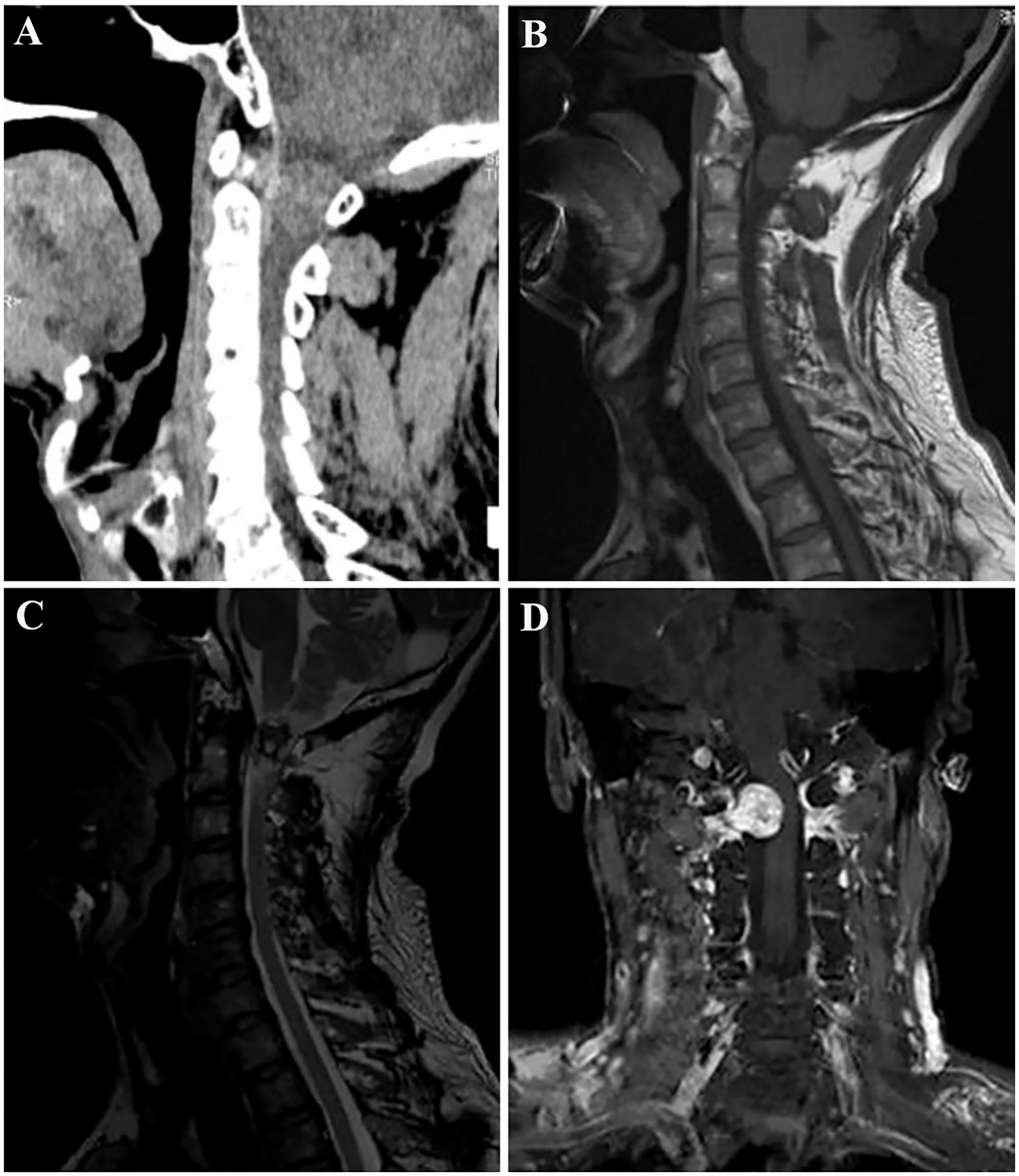
Spine computed tomography (CT) demonstrated a slightly hyperdense nodule lesion with a little spot low and high density of the C1-2 level, and well-circumscribed without obvious bone destruction **(A)**. Contrast-enhanced magnetic resonance imaging (MRI) of the spine; the lesion was isointense on T1-weighted images **(B)**, and hypo- to hyperintense on T2-weighted images **(C)**. After intravenous administration of gadolinium, the tumor showed obvious peripheral enhancement, presenting a growing tendency along the right second intervertebral foramen and compressing the spinal cord to the left side **(D)**.

Plain X-ray revealed a straight and hyper-osteogenic cervical spine without bone destruction. A spine CT scan showed a slightly hyperdense nodule lesion with a little spot low and high density of C1-2 level, well-circumscribed without evident destruction of the surrounding bone (Figure 1A). The CT value was ~46 HU. Magnetic resonance imaging (MRI) of the spine showed that the mass was 20 mm × 16 × 17 mm, isointense onT1-weighted images (T1WI), and hypo- to hyper-intense on T2-weighted images (T2WI) of the intradural extramedullary tumors at the C1-2 level (Figures 1B,C). After intravenous administration of gadolinium, the mass showed obvious enhancement, presenting a growing tendency along the right second intervertebral foramen and compressing the spinal cord to the left side, without bone and soft tissue destruction (Figure 1D). Then, the diagnosis of schwannoma at the C1-2 level was made before surgery based on the above imaging findings.

### Surgical Findings and Pathological Examination Results

**Figure 2:**
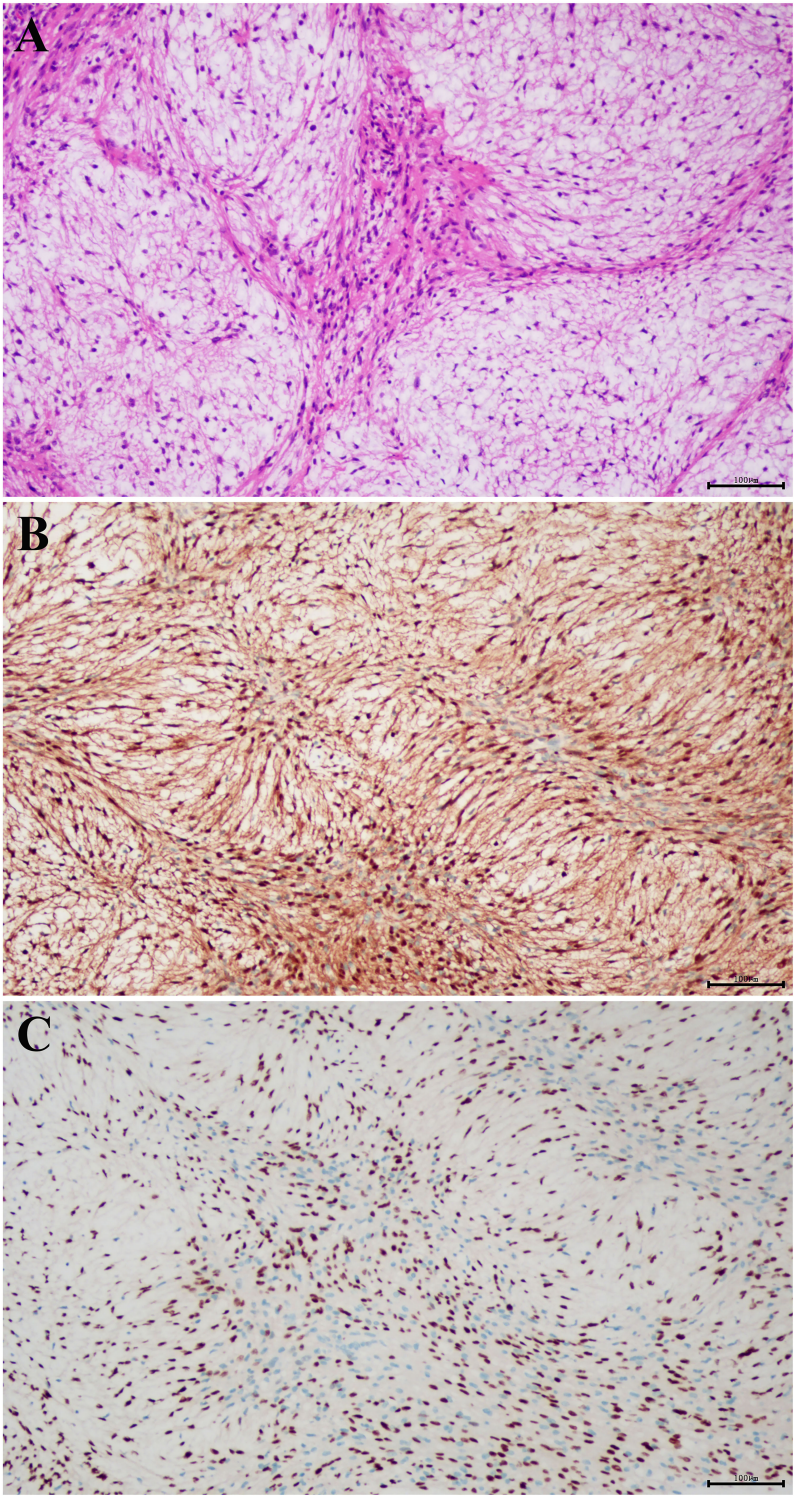
Histopathological examination showed that the lesion was grayish red, well-circumscribed, with average blood supply, soft in quality, and located in the intraspinal subdural region **(A)** (hematoxylin and eosin staining; magnification, × 100). The tumor cells were positive for **(B)** S-100 and **(C)** SOX10 (magnification, × 100).

Based on the radiological diagnosis, complete excision at the C1-2 level was performed through the posterior cervical approach. First, the skin around the C1-2 cervical vertebra was incised, and then its right lamina was removed. Intraoperatively, the tumor was found in the intraspinal subdural region, and it was a grayish red, soft, well-circumscribed mass with an average blood supply. The lesion originated from the C2 cervical nerve root. The nerve root sheath migrated into the capsule of the tumor, and it was difficult to separate the tumor from the nerve root, so the C2 cervical nerve root was dissected and separated along the edge of the tumor. Finally, the tumor was completely removed, and intraoperative monitoring showed that nerve function was normal. The mass was about 20 × 16 × 17 mm in size. Histopathological examination of the lesions revealed characteristic findings. Histopathological examination showed that the lesion was grayish red, soft, well-circumscribed with average blood supply, and located in the intraspinal subdural region (Figure 2A). Immunohistochemical analysis revealed that the tumor cells were positive for S-100 protein (Figure 2B) and SOX10 (Figure 2C), and negative for SMA, CD34, and STAT6. The final pathological diagnosis of the lesion was nerve sheath myxoma.

### Postoperative Course

The patient recovered well and was discharged in a few days in satisfactory condition after the operation. Postoperative MRI re-examination revealed no obvious residual or recurrence 2 weeks after the surgery (Supplementary Figure 1). The patient was followed up by telephone half a year after the operation, and the numbness of his left finger obviously disappeared.

## Discussion

Nerve sheath myxoma is an unusual benign peripheral nerve sheath-derived neoplasm in the dermal layer of the skin (6). This tumor is similar with neurothekeoma in cytological and histological pathology. Most studies have argued that histogenesis originates from Schwann cells or undifferentiated nerve sheath precursor cells. Ordinarily, nerve sheath myxoma is a slow-growing, solitary, superficial, multinodular, and painless tumor in the 5- to 25-mm size range, commonly seen in 30–40 years old women (2). Nerve sheath myxoma mainly occurs in the upper extremities or head-neck region, but it is exceedingly rare in intraspinal canals. To the best of our knowledge, only eight cases of intraspinal nerve sheath myxoma have been reported, including two cases in the thoracic spine, four cases in the lumbar spine, and two cases in the thoracolumbar spine. Notably, our case is the first in the cervical spinal canal.

We reviewed all the cases of nerve sheath myxoma in the spinal canal in the reported literature (Table 1). Of the 9 cases, there were six males and three females with an age range of 31-84 years. This characteristic of female dominance is inconsistent with the report Fetsch (2). The authors suggest that it is related to our small sample size, which needs to be analyzed and summarized by large samples later. There was one case in the cervical spine, two cases in the thoracic spine, four cases in the lumbar spine, and two cases in the thoracolumbar spine. Of these cases, six cases had MR images. In this case report, we first give a comprehensive description of nerve sheath myxoma, including pathology, CT and MR findings, and clinical treatment.

**Table 1:** Summary of previously reported cases of intraspinal nerve sheath myxoma.

**Authors**	**Age/Sex**	**Location**	**Size**	**T1WI**	**T2WI**	**Enhanced T1WI**	**Treatment**	**Outcomes**
Paulus et al. (3)	32/F	L4-5	8 × 10 × 20 mm	NA	NA	Peripheral enhancement	NA	NA
Paulus et al. (3)	47/M	T6	25 × 20 × 20 mm	NA	NA	NA	Total resection	No recurrence
Kaar et al. (4)	84/F	T12-L1	22 × 15 × 7 mm	NA	Hyperintensity	Obvious enhancement	Subtotal resection	NA
Lee et al. (5)	64/M	L1-2	15 × 10 mm	NA	NA	Obvious enhancement	Subtotal resection	NA
Lee et al. (5)	31/M	L2	23 × 20 mm	NA	NA	NA	Total resection	NA
Onoprienko et al. (6)	52/F	L1	20 × 5 mm and 10 × 10 mm	NA	Hyper-to Isointensity	Obvious enhancement	Total resection	No recurrence
Yamato et al. (7)	74/M	T8	19 × 11 × 9 mm	Hypointensity	Hyperintensity	Peripheral enhancement	Total resection	No recurrence
Yamato et al. (7)	58/M	T12-L1	19 × 15 × 9 mm	Hypo-to isointensity	Hyper-to isointensity	Peripheral enhancement	Total resection	No recurrence
Present case	57/M	C1-2	20 × 16 × 17 mm	Isointensity	Hypo- to hyperintense	Peripheral enhancement	Total resection	No recurrence

*F, female; M, male; NA, not available*.

Nerve sheath myxoma of the spine usually manifests as a well-demarcated, slightly hyperdense nodule lesion without bone destruction on CT. Up to date, calcification in lesions has not been reported in the literature. The size of tumor is usually 5–25 mm, and is related to the location of the lesion in the spinal canal, and tumor growth is restricted. Generally, there are no obvious clinical symptoms. The patients were usually admitted by the doctors because of the compression to the peripheral nerve and spinal cord caused by the mass. The lesion may appear hypo-to isointensity signal in contrast to the surrounding skeletal muscle on T1WI, high and heterogeneous signal on T2WI, and peripheral enhancement on contrast-enhanced T1WI. In our case, the lesion was hypo-to hyperintense on T2WI, which was inconsistent with the imaging findings previously reported by Yamato (7). The signal intensity on T2WI depends on the amount of mucus components, and the edge of the lesion can be significantly enhanced (7). In addition, cystic necrosis and bleeding are rarely seen within the tumor. The lesion is located in the extramedullary dura. As the lesion grows, it may compress the spinal cord and cause enlargement of the adjacent foramina.

Nerve sheath myxoma is a myxoid variant of schwannoma (2). The diagnosis of nerve sheath myxoma is based on histological pathology, and immunohistochemical pathology is not useful for the differentiation between nerve sheath myxoma and schwannoma. Nerve sheath myxoma and neurothekeoma are two similar benign skin tumors originating from the dermis that are difficult to be distinguished clinically (8). Currently, the two tumors can only be distinguished by histomorphology and immunohistochemistry. Sheth et al. (9) found that the former is derived from peripheral nerve sheath cells, and the latter is derived from fibroblasts. Histopathological examination revealed multiple mucous nodules of varying sizes scattered between the fibrous compartments of nerve sheath myxoma (10). In addition, the immunohistochemical findings of these two tumors are different. Nerve sheath myxoma is positive for S-100 and glial fibrillary acidic protein (GFAP), and negative for epithelial membrane antigen (EMA), while neurothekeoma is just the opposite (11, 12).

Clinically, surgical resection is the primary method. Most of the tumors can be removed entirely, except for lesions that are in close adhesion to the medullary cone and multiple nerve roots. Postoperative auxiliary treatment is not required. Malignant transformation or metastasis has not been reported. Jason L. Hornick reported that the local recurrence rate was 14%, so it is necessary to follow up closely after operation (13).

Although nerve sheath myxoma is rare, it typically shows a well-circumscribed mass with hypo-to iso intensity on T1WI, heterogeneous intensity on T2WI, peripheral obvious enhancement, presenting a growing tendency to the intervertebral foramen. The differential diagnosis of nerve sheath myxoma should be considered. Surgical resection is regarded as the best treatment for nerve sheath myxoma. The final diagnosis of nerve sheath myxoma should be confirmed by pathological examinations, including histopathological and immunohistochemical analyses.

## Data Availability Statement

The original contributions presented in the study are included in the article/Supplementary Material, further inquiries can be directed to the corresponding author.

## Ethics Statement

The studies involving human participants were reviewed and approved by the Ethics Review Committee of the Second Affiliated Hospital of Zhejiang University School of Medicine. The patients/participants provided their written informed consent to participate in this study. Written informed consent was obtained from the individual(s) for the publication of any potentially identifiable images or data included in this article.

## Author Contributions

CW performed the data acquisition. CW, FX, QL, FD, and LL performed the radiological images analysis. FX, YJ, and QC performed the manuscript preparation. All authors contributed to the article and approved the submitted version.

## Funding

This research was supported by the Zhejiang Provincial Natural Science Foundation of China under Grant No. LY21H180003.

## Conflict of Interest

The authors declare that the research was conducted in the absence of any commercial or financial relationships that could be construed as a potential conflict of interest.

## Publisher's Note

All claims expressed in this article are solely those of the authors and do not necessarily represent those of their affiliated organizations, or those of the publisher, the editors and the reviewers. Any product that may be evaluated in this article, or claim that may be made by its manufacturer, is not guaranteed or endorsed by the publisher.

## Supplementary Material

Supplementary Figure 1 Sagittal T2-weighted image (WI) showed no signs of recurrence or metastasis in the spinal canal 2 weeks after surgery.
