# Supplementary figures and images for: Intradural Extramedullary Nerve Sheath Myxoma of the Cervical Spine: A Case Report and Review of Literature
Source: Front Surg. 2022 Jan 7;8:722254. doi: 10.3389/fsurg.2021.722254 (PMC8776648; doi:10.3389/fsurg.2021.722254)

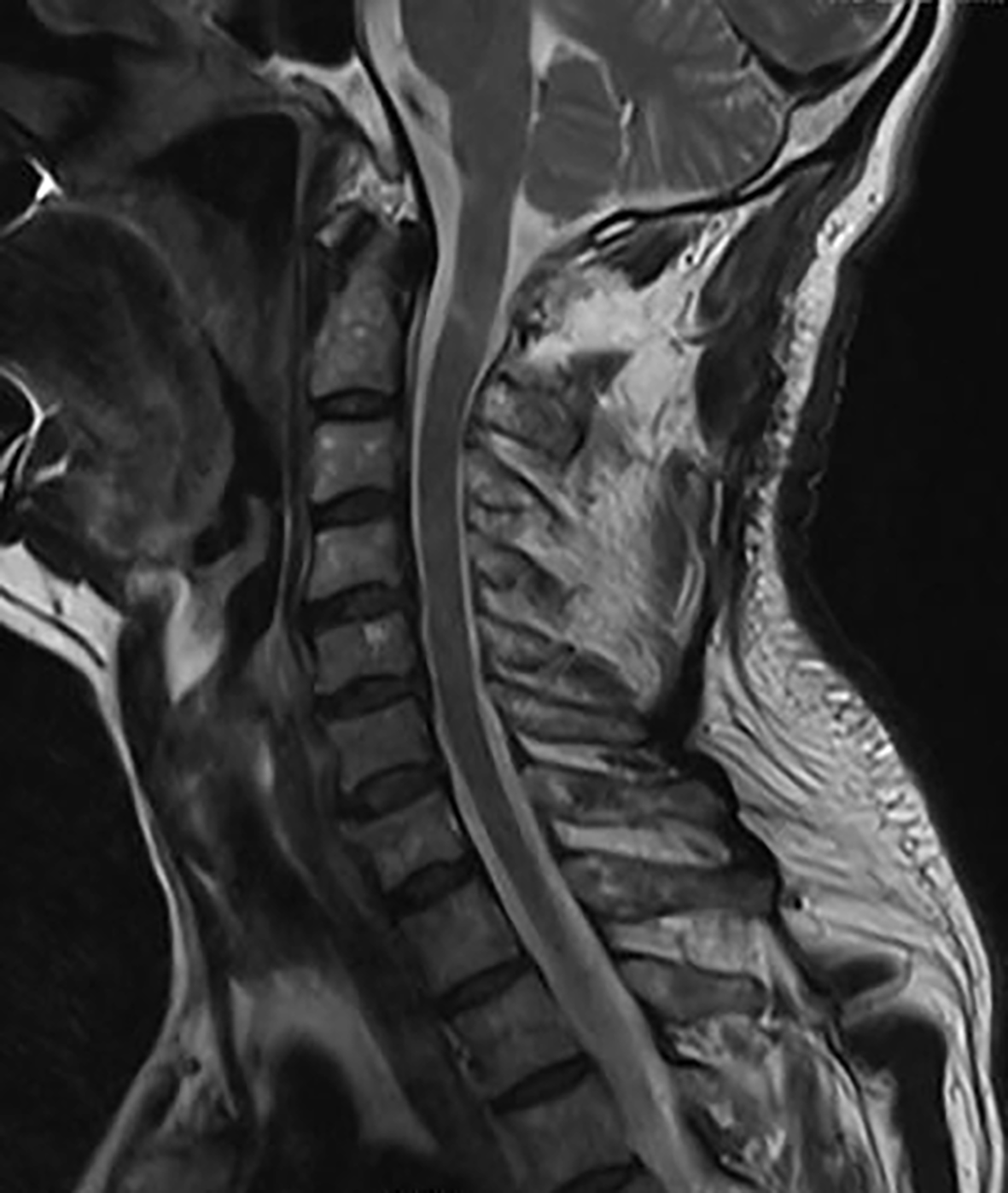

Supplement: Supplementary Figure 1 — Sagittal T2-weighted image (WI) showed no signs of recurrence or metastasis in the spinal canal 2 weeks after surgery. [file Image_1.TIF]
